# Influence of Convective and Vacuum-Type Drying on Quality, Microstructural, Antioxidant and Thermal Properties of Pretreated *Boletus edulis* Mushrooms

**DOI:** 10.3390/molecules27134063

**Published:** 2022-06-24

**Authors:** Miruna Popa, Ioan Tăușan, Olga Drăghici, Amalia Soare, Simona Oancea

**Affiliations:** 1 Department of Agricultural Sciences and Food Engineering, “Lucian Blaga” University of Sibiu, 7–9 Dr. Ion Ratiu Str., 550012 Sibiu, Romania; florentina.popa@ulbsibiu.ro (M.P.); olga.draghici@ulbsibiu.ro (O.D.); 2 Department of Environmental Sciences, “Lucian Blaga” University of Sibiu, 5–7 Dr. Ion Ratiu Str., 550012 Sibiu, Romania; ioan.tausan@ulbsibiu.ro; 3 Energy Department, National Research and Development Institute for Cryogenic and Isotopic Technologies-ICSI, 4 Uzinei Str., 240050 Râmnicu Vâlcea, Romania; amalia.soare@icsi.ro

**Keywords:** *Boletus edulis* puree, hot air drying, centrifugal vacuum drying, freeze drying, pretreatment, antioxidant, color, ATR-FTIR, SEM, DSC

## Abstract

Freshly harvested *Boletus edulis* mushrooms are subjected to rapid loss of quality due to the high moisture content and enzymatic activity. Drying time, quality characteristics, microstructural and thermal properties were studied in mushrooms ground to puree subjected to hot air drying (HAD), freeze drying (FD) and centrifugal vacuum drying (CVD). The influence of hot water blanching and UV-C pretreatments was additionally investigated. The rehydration ability of mushroom powders was improved by FD, especially without pretreatment or combined to UV-C exposure. The HAD and CVD, with no pretreatment or combined to UV-C, ensured good preservation of phenolics and antioxidant activity of dried mushrooms. The total difference in color of mushroom pigments extracted in acetone was lower in samples dried by CVD and higher in ones by FD. Blanching before HAD produced whiter product probably due to the reduced polyphenoloxidase activity. Scanning Electron Microscopy (SEM) analysis showed fewer physical changes in FD-samples. Heat-induced structural changes were noticed by Differential Scanning Calorimetry (DSC), Thermogravimetry (TG) and Derivative Thermogravimetry (DTG) analysis, in particular of biopolymers, confirmed by ATR-FTIR analysis. Based on our complex approach, the UV pretreatment of mushrooms could be a better alternative to water blanching. Centrifugal vacuum emerged as a new efficient drying method in terms of bioactive compounds, color and thermal stability, while FD led to better rehydration ability and microstructure.

## 1. Introduction

Wild-grown edible *Boletus edulis* mushrooms, also known as porcini, Steinpilz, king bolete or penny bun, are widely consumed and appreciated for their flavor, nutritional value, and extra health benefits as functional foods or ingredients, as well [1,2]. These mushrooms may be consumed as such or in a form of preservation commonly by thermal processing. Mushrooms are subjected to rapid loss of quality characteristics and bioactive compounds due to their high moisture content (87–95%), respiratory rate and polyphenoloxidase activity [3], the recommendation being that fresh mushrooms be stored at 20–25 °C up to three days or at 4 °C for seven days [4]. Preservation strategies become essential for assuring the consumption of *B. edulis* not only seasonally (summer-autumn) but throughout the year. The appropriate preservation technique either thermal or physical-chemical [5], should be evaluated for each type of mushrooms. The thermal treatment by drying is one of the most common preservation methods used for extending the shelf life of mushrooms. The conventional drying methods involve heating (solar, convective, conductive, radiative, dielectric) being operated individually or in combination [6]. When using such techniques, the optimization of process parameters (temperature, time, air velocity, etc.) should be applied to produce a minimal impact on the quality of the final product. It has been shown that air and freeze drying of mushrooms produces little or no change in the proximate composition of *B. edulis*, and the final dried products can be stored up to 12 months at 4 °C or 20 °C [7]. However, freeze drying (lyophilisation) has been considered an expensive method, being usually employed for high-value products [8] despite it being one of the most product-friendly method based on vacuum drying without thawing.

Hereby, we have investigated for the first time a thermal method of centrifugal vacuum drying of *B. edulis* mushrooms, also known as speed-vacuum drying, which represents a less expensive alternative of drying based on the combination of vacuum drying and centrifugation. Every single drying method has advantages and limitations, related either to the final product (loss of nutrients and bioactive compounds, color change, texture modification, rehydration capacity) or to the applied technology (energy cost, time and sample amount requirements), so that each technique should be individually assessed in relation to the specific starting food material. Most studies investigating the drying process of various mushrooms reported results obtained from the dehydration of mushroom slices, whose thickness highly influence the process among other input parameters [9]. By our knowledge, no reported scientific study on drying the mushroom puree has been found, industrial processing involving the drying of slices of mushrooms. With regard to mushroom puree, the study of Pasban et al. [10] reported that xanthan or cress seed gums added to white button mushroom puree were suitable for obtaining a good, stabilized mushroom foam which may be further processed, e.g., by drying, but without reporting the drying application. However, pureeing has been considered an efficient pretreatment in terms of reduced drying time or increased bioactive content of various food (chokeberry, strawberry, orange, kiwi, cranberry, kale, banana, papaya), being applied before different drying technologies, such as freeze drying [11] or refractance window technology [12]. Such application guided us to conduct drying on mushroom puree.

Pretreatments of fresh mushrooms prior to drying, using physical thermal or non-thermal methods (water/steam blanching, microwave, pulsed electric field, gamma or UV irradiation, ultrasounds, high hydrostatic pressure) or chemical processes (soaking in chemical solutions) is often practiced, in order to reduce the numbers of contaminating microorganisms, to inactivate the enzymes responsible for the browning reactions or to positively influence the drying process [13,14,15]. An inadequate pretreatment may negatively influence the quality of the final mushroom product, due to nutrient loss, reduction of the level of antioxidant compounds or antioxidant activity, sensory/color changes or rehydration ability.

Considering that knowledge of appropriate preservation technology is essential to define product quality, the present study aimed to investigate the drying behavior of *B. edulis* mushroom subjected to three methods of drying, convective and vacuum, the effects of thermal and non-thermal pretreatments and drying type on the rehydration ratio, phenolic content, antioxidant activity, color and thermal properties. The studies were completed by ATR-FTIR and SEM analysis of mushroom powders. Distinctively from other published works, we have tested drying of mushrooms in the form of blend/puree and applied for the first time the centrifugal vacuum as drying method.

## 2. Results

### 2.1. Drying Behavior of B. edulis Mushrooms Subjected to Various Methods of Pretreatment and Drying

Physical thermal and non-thermal pretreatments of mushroom samples by blanching and UV-C irradiation respectively were applied prior to drying in order to control the quality of the final products (microbial load decrease, enzyme inactivation, toxic elements removal or color retention) [16]. Our previous results showed that exposure of *Boletus* dried mushrooms to UV-C light contributed to a good retention of antioxidant compounds of polyphenolic structure [15].

Three types of drying of *B. edulis* mushrooms were performed in order to obtain mushroom powders useful as food/functional ingredient: hot air drying (HAD), freeze drying (FD) and centrifugal vacuum drying (CVD), the latter for the first time hereby applied. In contrast to most published studies dealing with mushroom drying, we conducted such processes on mushroom blend/puree samples instead of mushroom slices. All the applied methods enable the control of the main process parameters; thus the drying process is reproducible.

Our results showed that both applied pretreatments (conventional hot water blanching and UV irradiation) did not influence the HAD total time but led to an increase of ~54% of the total drying time by CVD in order to reach ~7% moisture, compared to control (without pretreatment). The initial moisture content of all pretreated samples was slightly higher (~87%) than that of control (84.585%), which influenced the drying characteristics. Most published studies on pretreated fruits, vegetables or herbs, in particular by blanching, showed a reduction of the drying time. However, results may vary significantly indicating even lower drying rates in relation to the product, the type of pretreatment and subsequent drying processes [14,17]. The study of Argyropoulos et al. [2] on dried slices of *B. edulis* mushrooms showed no effect of water and steam blanching, as well as chemical pretreatment, on the total time of drying by HAD compared to untreated samples. Similarly, other results on button mushrooms (*Agaricus bisporus*) confirmed that HAD times of blanched whole mushrooms did not statistically shorten as compared to untreated ones [18]. Due to the lack of studies on CVD time in relation to water blanching or UV pretreatment of mushrooms or other food products, the comparison of our results could not be made for these results.

**Figure 1:**
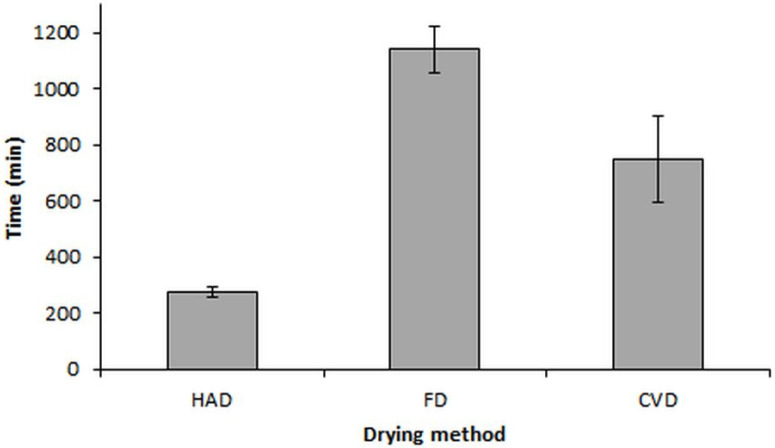
Total drying time of *B. edulis* samples under different drying methods to reach moisture content of 6.55% (HAD), 6.31% (FD) and 7.78% (CVD).

The different drying methods had significant effects on the total drying time required to reduce the moisture content from 86.585–87.066% to ~8%, irrespective of the applied pretreatment, as shown in Figure 1. FD required the longest drying time (19 h), while HAD determined the shortest time (275 min). These results are in accordance with other published data on food products [19]. The effect of pretreatment on the total drying time of investigated samples, irrespective of the drying method, showed a time increase (1.39 ÷ 1.49-fold) for UV-pretreated and blanched mushrooms, respectively.

By carrying out linear regression on experimental data for samples dried by HAD and CVD, considering drying time as predictor variable and pretreatment and drying methods as response (dependent) variables, we found significant regressions between these variables. Control samples did not undergo any pretreatment except drying. The obtained results (regression coefficients, F-statistic, degree of freedom and *p* values) are given in Table 1.

Very strong relationships were found between the HAD method and drying time of pretreated and control samples. In the case of CVD method, strong relationships were found for all the pretreatment methods. Yet, the values of the regression coefficients are slightly lower than those for the HAD method. Regarding the CVD method, a logarithmic regression model fits for all types of pretreatment methods, showing a logarithmic decrease of the moisture content with drying time. The model is significant, yet the relationship is weak (*p* = 0.0017, r^2^ = 0.249, F = 11.66, df = 31).

### 2.2. The Effects of Pretreatment and Drying Methods on the Rehydration Ratio, Phenolic Content and Antioxidant Activity

**Figure 2:**
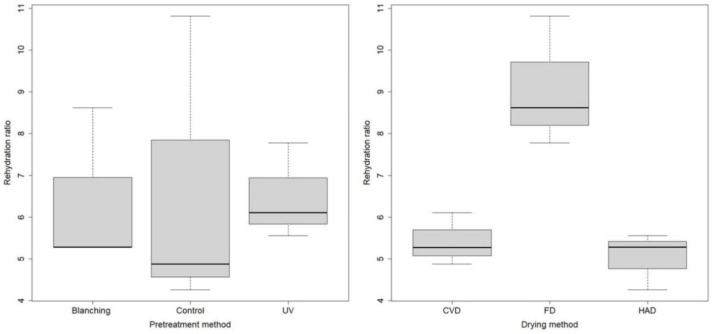
Boxplot of the rehydration ratio of *B. edulis* dried mushrooms in relation to pretreatment for all dried samples, and in relation to drying for all pretreated samples.

To further investigate the influence of different pretreatment and drying methods on the structural quality properties of dried *B. edulis* mushrooms, the rehydration ratio of the final products have been evaluated. The higher the rehydration ratio, the better the quality of the product. The values of the rehydration ratio varied from 4.259 to 10.819 for different operating conditions. As shown in Figure 2, there are no statistically significant differences between the rehydration ratio of dried mushrooms and the different pretreatment methods (ANOVA *p* = 0.992, F = 0.009, df = 2). This statistical analysis was performed on experimental data for samples dried by all studied drying technologies. However, UV pretreatment seems to improve the rehydration ratio, especially when combined with FD (7.77 ± 0.09). Instead, statistically significant differences were found for rehydration ratio according to the drying methods (ANOVA *p* = 0.0061, F = 13.39, df = 2). This statistical analysis was performed on experimental data for samples pretreated by all studied pretreatments. Moreover, applying the Tukey post-hoc test, statistically significant differences between FD and HAD methods (p-adj = 0.008014) and between FD and CVD methods (p-adj = 0.012901) were noticed. The rehydration ability of dried mushrooms was improved by using the FD method compared to HAD and CVD which produced similar rehydration ratios of lower values. The values of the rehydration ratios of the freeze-dried samples varied from 8.61 to 10.81 according to the applied pretreatment. This efficacy of rehydration is related to the porous structure created by solid water during FD [8]. Other researchers reported similar rehydration ratio values for freeze-dried *A. bisporus* mushrooms (7.87 ± 0.08) compared to air dried ones at 60 °C (3.17 ± 0.06) [20], values of 5.45 ± 0.107 for freeze-dried shiitake mushrooms (*Lentinula edodes*) [21] or substantially higher rehydration capacity of freeze-dried *B. edulis*, *Pleurotus ostreatus*, *Pleurotus sajor-caju*, *Coprinus comatus* than hot air dried samples [7,22,23,24].

Regarding the rehydration ratio, FD with no pretreatment or combined to UV-C exposure as non-thermal pretreatment is a good choice of mushroom processing.

The fruiting bodies of *B. edulis* mushrooms contain various antioxidant compounds, such as polyphenols. Our present investigation showed that the total phenolic content (TPC) expressed as mg gallic acid equivalents (GAE) per 100 g of dry weight (DW) of the ethanol extracts obtained from the raw *B. edulis* untreated samples stored at −70 °C, was 1319.227 ± 33.285 mg GAE/100 g DW for control (no treatment), slightly lower than that of the UV pretreated sample (1387.091 ± 41.115 mg GAE/100 g DW), the results being in accordance to our previously published paper [15]. The TPC of control sample was significantly higher than that of the blanched sample (459.391 ± 12.421 mg GAE/100 g DW). Our findings regarding the TPC of fresh wild *B. edulis* mushrooms are similar to other reported ones based on comparable analysis approach, e.g., 1277.5 mg GAE/100 g DW [25], 1250 mg GAE/100 g DW [26], despite that lower [27] or greater contents have also been reported [28,29].

The average TPC of raw samples were 14–18% higher than that of the corresponding dried samples by HAD, FD or CVD. A decrease of the TPC of *B. edulis* mushrooms by FD was found by other researchers but an increase by air drying [7].

**Figure 3:**
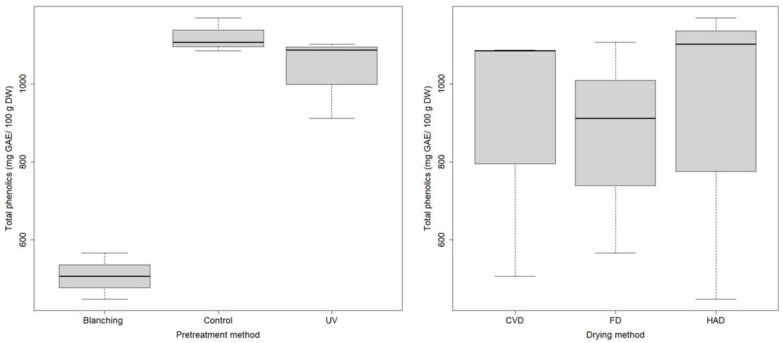
Boxplot of the TPC of *B. edulis* dried mushrooms in relation to pretreatment for all dried samples, and in relation to drying for all pretreated samples.

The results regarding the evaluation of the phenolic content of dried mushrooms in relation to the pretreatment and drying method are presented in Figure 3.

The statistical analysis confirmed the significant differences of TPC of dried samples in relation to the pretreatment (ANOVA *p* = 0.00011, F = 59.7 df = 2). This statistical analysis was performed on experimental data for samples dried by all studied drying technologies. Moreover, applying the Tukey post-hoc test, significant differences of TPC were observed in dried mushrooms between control (no pretreatment) and blanched samples (p-adj = 0.00013), and between blanched and UV-pretreated samples (p-adj = 0.00031), showing that non-thermal UV-C exposure of mushrooms may be an efficient alternative to other physical pretreatments. The significant loss of phenolic compounds probably due to leaching during hot water blanching has also been observed by other authors [30].

Among different drying methods, no statistically significant differences were found in relation to TPC. This statistical analysis was performed on experimental data for samples subjected to all studied pretreatments. The control sample dried by HAD registered the highest TPC (1169.602 ± 17.325 mg GAE/100 g DW), while the blanched sample dried by HAD showed the lowest value (447.617 ± 5.121 mg GAE/100 g DW). HAD determined a slightly higher TPC by 5% compared to FD, despite that FD has been generally considered a proper method for preserving several bioactive compounds including high-molecular-weight polyphenols, compared to heat-drying methods. However, in the literature there are studies that suggest reconsideration of freeze drying in relation to pharmacological properties of medicinal plants [31]. A study on dried *B. edulis* mushroom found that FD caused a slight reduction of the level of polyphenols compared to air drying at temperature gradients of 40 and 60 °C for 15 h [7].

Regarding the total antioxidant activity (TAA) as measured by the Ferric Reducing Antioxidant Power (FRAP) assay, and expressed as mg ascorbic acid (AA) per 100 g of dry weight (DW), our results showed that the average TAA of ethanol extracts obtained from the raw *B. edulis* mushrooms stored at −70 °C (720.039 ± 14.575 mg AA/100 g DW) were 24–28% higher compared to the corresponding dried samples by HAD, FD or CVD. Similar to TPC, the obtained TAA of raw untreated samples (864.631 ± 18.137 mg AA/100 g DW) was lower than that of the UV pretreated sample (940.316 ± 15.468 mg GAE/100 g DW), which is in accordance to our previously published paper [15], but higher than that of the blanched sample (355.171 ± 10.120 mg GAE/100 g DW). These results indicate that the UV-C irradiation of wild *B. edulis* mushrooms is a better choice for pretreatment while blanching leads to a considerable loss of the antioxidant activity, due to high temperature involved.

**Figure 4:**
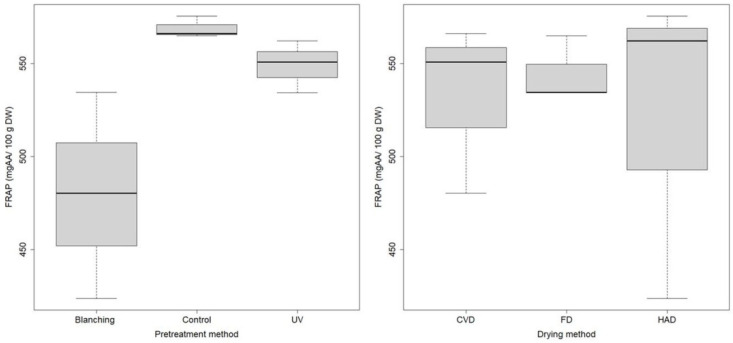
Boxplot of the TAA of *B. edulis* dried mushrooms in relation to pretreatment for all dried samples, and in relation to drying for all pretreated samples.

The results regarding the evaluation of the antioxidant activity of dried mushrooms in relation to the pretreatment and drying method are presented in Figure 4.

The pretreatment method highly influences the TAA of dried samples. Statistically significant differences of TAA in relation to the pretreatment method were found (ANOVA *p* = 0.0369, F = 6.014. df = 2). This statistical analysis was performed on experimental data for samples dried by all studied drying technologies. Moreover, applying the Tukey post-hoc test, statistically significant differences among dried samples subjected to different pretreatments were observed, such as between the control and blanched samples (p-adj = 0.0376) and marginal significant differences between blanched and UV samples (p-adj = 0.093).

### 2.3. Color Changes in Extracts of Pretreated Dried Mushrooms

The color characteristics of the raw and the pretreated dried (HAD, FD and CVD) *B. edulis* mushrooms were measured based on pigments soluble in acetone using the software of the spectrophotometer according to DIN EN ISO 1164 (xyz, CIE, L*a*b) and ASTM E 313 (yellowness and whiteness indices). The color characteristics using the CIELAB system (luminosity L*, red-green a*, yellow-blue b*, color differences ΔE) and ASTM method E313 (whiteness and yellowness indices) are presented in Table 2.

All applied pretreatment and drying methods led to color changes in the mushroom powders compared to the raw samples.

The values of lightness/darkness (L*) changed in relation to the type of pretreatment and drying. Blanching and UV exposure of mushrooms determined an increase in the lightness either in raw or dried samples. The HAD and CVD drying methods led to an increase in the darkness of the untreated mushrooms, while untreated FD-samples were brighter than the raw untreated ones. Blanching before drying determined a slight decrease of L* value in case of CVD method and a slight increase in case of HAD and FD methods compared to the values obtained for the raw blanched sample. Similar results of lighter colors of freeze-dried *A. bisporus* mushrooms firstly subjected to water blanching has been reported [32]. Other authors showed a decrease in the lightness of *B. edulis* mushroom slices subjected to chemical pretreatment and water and steam blanching [2]. However, authors concluded a remarkable stability of lightness of the pretreated samples during drying using hot air (50 °C, 60 °C and 70 °C). The distinct reported results may be related to the different color evaluation technique [33], the present approach being based on the acetone extraction of pigments and not surface reflectance method used for mushroom slices. The UV exposure followed by drying process did not significantly influence the L* values of samples dried by CVD and FD, compared to HAD which determined darker colors. As shown in Table 2, all samples displayed colors from yellow-yellow to greenish-green (negative a* and positive b*), with the exception observed for the control HAD- and CVD-dried samples, which exhibited positive values of both a* and b* characteristics (red-orange-yellow). The calculated red-green differences Δa* observed for dried samples according to various pretreatment and drying methods showed positive values, indicating a deeper red chroma due to browning reactions. Concerning the yellow-blue difference Δb*, all samples were found bluer than their corresponding reference (negative values) with the exception observed for the control CVD-dried mushrooms, which exhibited positive value indicating samples yellower than the reference (raw untreated). The total difference in color (ΔE*) ranged from 17.41 to 52.98, depending on the pretreatment and drying method. The values indicate greater color changes in control FD-dried mushrooms, followed by blanched HAD-dried and UV-FD-dried samples. Drying by CVD method, in particular for untreated and blanched samples, caused the least color changes. However, considering the mean ΔE values, the least color changes were noticed for UV pretreated samples in relation to the pretreatment method, while the least color changes were observed for CVD-dried samples with respect to the drying method.

The whiteness index of mushrooms increased during the pretreatment and drying processes, the least change being registered in control CVD-dried samples while the greater one in the blanched HAD-dried sample which produced whiter product probably due to the involved temperature (60 °C) which reduced the polyphenoloxidase activity responsible for browning reactions [32]. The yellowness index decreased in all dried samples subjected to different pretreatments (blanching, UV) or drying processes (HAD, CVD, FD). Untreated raw samples and CVD-dried samples showed the highest yellowness indices, probably due to the greater enzymatic activity.

### 2.4. Microstructural Properties of Mushroom Powders by SEM Analysis

**Figure 5:**
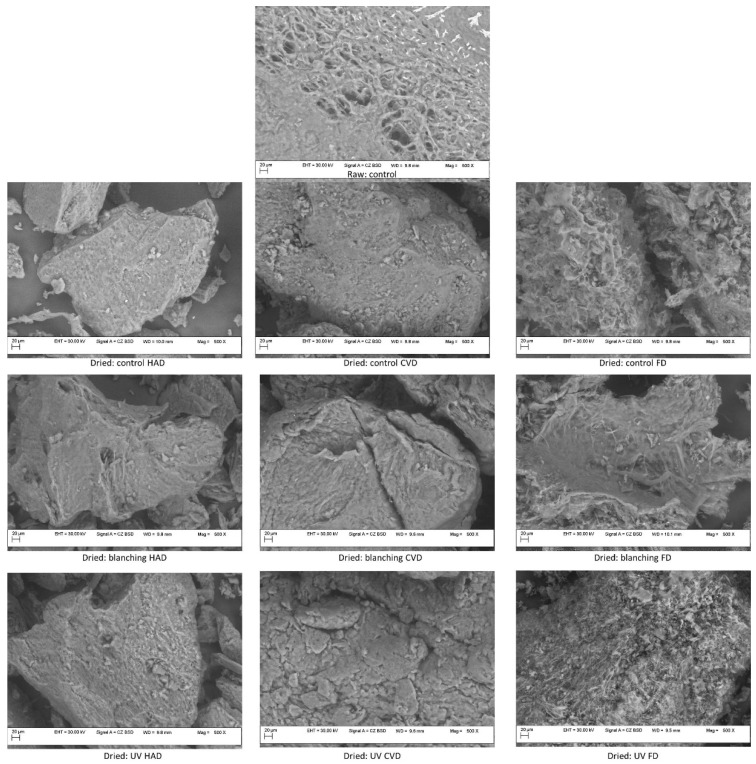
The SEM micrographs of raw and dried *B. edulis* mushrooms under different pretreatment and drying methods (500×).

The microstructure of mushroom raw and powder samples subjected to different pretreatment and drying procedures illustrated in Figure 5 was observed under the “Variable Pressure” mode using the Backscattered Electron Detector (BSD). This detector allows identifying different phases or distribution of elements throughout the sample depending on their atomic number: the higher the Z number, the lighter the area due to the strong signal. A rather homogeneous distribution of elements was noticed from the micrographs. The thermal treatment determined physical changes in the sample microstructures due to the disruption of cell walls which determined a disordered structure [33]. The images of all samples show breakages, cracks and holes due to the grinding process causing the breakage of intermolecular bonds. A homogenous compact structure was observed in samples pretreated by UV exposure before drying. Among different types of drying, freeze drying, in particular in untreated or UV-treated samples, led to the fewest physical changes compared to the raw control samples, showing microporous and fibrous structures. Lewicki and Pawlak [34] confirmed that the microstructure changes of food samples subjected to drying are mostly due to the tissue thermal and hydro stress being noticeable by macro- and micro-alterations of size, shape and internal structure.

### 2.5. Evaluation of Chemical Changes in Dried Mushrooms Using the Fourier Transform-Infrared (FTIR) Spectroscopy

**Figure 6:**
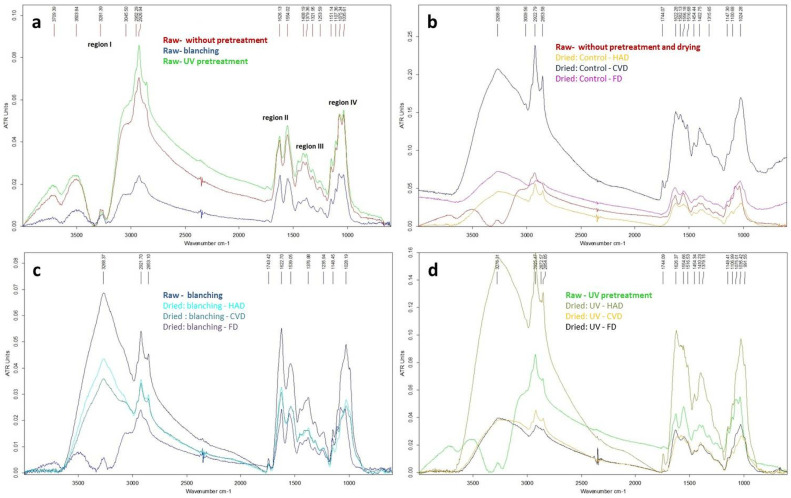
ATR-FTIR spectra of *B. edulis* mushrooms, subjected to different pretreatment and drying methods; (**a**) raw without/with pretreatment, (**b**) raw without pretreatment and drying/dried without pretreatment, (**c**) blanched raw/blanched dried, (**d**) UV-pretreated raw/UV-pretreated dried.

In this study, a reflectance FTIR spectroscopy (ATR-FTIR) analysis was performed for raw *B. edulis* samples stored at −70 °C (without pretreatment and subjected to pretreatments by blanching and UV) and for the corresponding dried samples. The obtained spectra are presented in Figure 6.

The FTIR spectra of the raw samples of mushrooms show four regions (Figure 6a), as follow [35]: (I) **Region I 3700-2800**, containing the following absorption bands: 3500–3700 cm^−1^ medium sharp bands due to stretching of free alcohol O-H, N-H stretching (3503 cm^−1^), 3281 cm^−1^ weak band alcohol O-H stretching intramolecular bonded, 3064–2854 cm^−1^ strong bands assigned to carboxylic O-H stretching (broad), amine N-H stretching, C-H stretching (CH_3_, CH_2_, CH) of acyl chains of lipids; (II) **Region II 1700-1500**, with medium-strong sharp two bands: the amide I band of proteins due to stretching of C = O (1626 cm^−1^), and the amide II band due to bending of N-H (1554 cm^−1^); absorptions in this region are also assigned to stretching vibrations of aromatic C = C in phenols or C = C of unsaturated fatty acids, as well; (III) **Region III 1500-1200** (proteins, phenols, polysaccharides and lipids), with 1455 cm^−1^, 1408 cm^−1^ due to bending/deformation of C-H (CH_2_, CH_3_ of alkanes chain and aldehydes); the peak at 1408 cm^−1^ is also assigned to the symmetric stretching vibration of COO^-^ in fatty and amino acids, 1379 cm^−1^ due to bending vibrations of phenol/alcohol O-H, C-O-H of carboxylic groups and C-H of aldehyde group (the latter associated with the weak band at 1734 cm^−1^ due to aldehyde C = O stretching), 1321 cm^−1^, 1253 cm^−1^ due to stretching of O-C of carboxylic acids and derivatives, phosphorus groups, sulphur C = S groups probably of ergothioneine, and C-N of amine, and bending of N-H assigned to amide III of proteins, as well (1253 cm^−1^); (IV) **Region IV 1200-1000** (mainly polysaccharides), due to stretching of C-OH of glycosidic bonds, C-O-C of pyranosyl rings of α- and β-glucans (1151 cm^−1^, 1107 cm^−1^), various carbohydrates/cellulose or due to stretching of PO_2_^−^ and C-O-P of phospholipids (strong bands at 1075 cm^−1^ and 1035 cm^−1^); absorption in this region may be also due to thiocarbonyl C = S of the amino acid ergothioneine.

Differences in the region I of absorption spectra were observed in dried mushroom samples without pre-treatment (Figure 6b), in particular for samples dried by HAD or CVD methods, where peaks at 2923 cm^−1^ and 2853 cm^−1^ attributed to C-H stretching of carbohydrates, were of higher intensity. Regarding the changes observed in the region II, additional peaks at 1744 cm^−1^ due to C = O stretching of phospholipids [36], 1582 cm^−1^, 1516 cm^−1^ (amide II, C-C stretching of phenyl ring, C-H bending) were evident in samples dried by CVD method. In the region IV, the peak at 1075 cm^−1^ disappeared in all dried samples without pretreatment, compared to control. All dried mushrooms displayed broad bands in the region IV compared to control (raw sample), probably due to heat-induced structural changes of carbohydrates. In dried samples pretreated by blanching or by UV irradiation (Figure 6c,d), an additional peak at 1744 cm^−1^ assigned to C = O stretching of phospholipids [36] was observed, with exception of sample pretreated by UV and dried by FD method. The drying method determined some structural changes in carbohydrates, as indicated by the differences registered in the absorption spectra region IV, compared to raw sample, where the peak at 1075 cm^−1^ is no more well-defined. The sample pretreated by UV irradiation followed by HAD showed notable changes in the region II of the IR absorption spectra (amide I and II), in particular for amide II band associated to N-H bending of secondary amides (the peak at 1554 cm^−1^ disappeared) and C-N stretching of peptide bond, probably due to protein conformational changes [37,38].

### 2.6. Thermal Properties and Mass Loss of Dried Mushrooms

The DSC investigation performed under N_2_ atmosphere was used to study the thermal behavior and postharvest quality of *B. edulis* mushrooms dried by various methods, while the TG analysis was carried out to determine the chemical mass changes.

**Figure 7:**
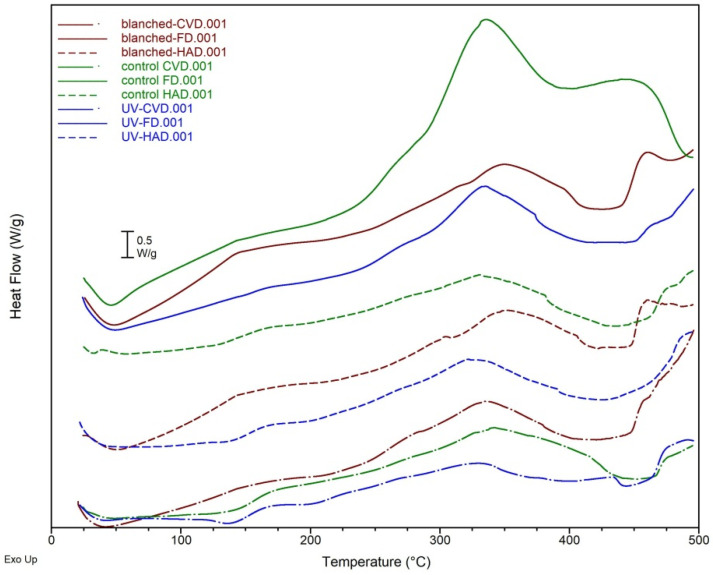
DSC thermograms of *B. edulis* mushrooms subjected to different pretreatment and drying methods for the temperature range of 25–500 °C.

The DSC thermograms and TG curves of dried samples using different drying methods (HAD, FD and CVD) and pretreatments (blanching, UV) are shown in Figure 7.

The DSC curves indicate five thermal transitions of dried mushrooms, of which 2 endothermic and 3 exothermic peaks. The broad first endothermic peak in the temperature range of 42–57 °C, observed in all DSC curves, except for that of UV-HAD sample, was mainly attributed to the gelatinization of polysaccharides [39]. Regarding other biomolecules such as proteins, their thermal behavior is complex due to the presence of hydrocolloids and other components in the mushroom matrix, so that DSC method may have limitations in such complex foods. However, proteins undergo gelation upon heating, consisting in endothermic transitions (denaturation, 50–85 °C) and exothermic processes (intermolecular aggregation), as studied on different types of proteins [40]. The lack of the first endothermic peak for the UV-HAD sample is correlated with changes associated with protein conformational modification observed by FTIR analysis.

As noted in Figure 7, an additional second endothermic event in the temperature range of 106–145 °C was predominant for samples (control, UV) dried by HAD and CVD methods. These events might be due to changes of chitin, a structural polysaccharide present in mushrooms [41,42,43]. Three exothermic peaks were further observed in DSC curves at temperature >105 °C. The first exothermic stage in the temperature range of 105–172 °C is probably due to melting of oligosaccharides and changes in other mushroom polysaccharides such as hemicellulose, as shown by other authors [43,44], as well as denatured protein aggregation [45]. The next two exothermic peaks at temperatures >250 °C correspond to the sample decomposition, pyrolysis of polysaccharides with generation of volatile substances and are in relation to significant weight loss in the TG curve. Considering that dried *B. edulis* mushrooms may be used as functional ingredients in various food products, heating of such products at temperatures >250 °C is not recommended due to the thermal decomposition.

The DSC-based values of thermal characteristics and the enthalpy change (ΔH) of the thermal transitions are presented in Table 3.

As shown in Table 3, the *T_p_* response of the mushroom powders for the first endothermic peak was affected by the drying conditions, showing higher values for HAD and FD. For the second stage of thermal transitions (P2), in the temperature range of 106–145 °C, mushrooms without pretreatment or previously exposed to UV-C and then dried by CVD showed higher values of *T_o_* and *T_p_* and ΔH of 34.78 J/g and 28.03 J/g, respectively. The lowest ΔH was found for control samples dried by HAD (6.18 J/g), indicating higher levels of changes in those biomolecules (polysaccharides, proteins) that remained native-like in the dried sample. The thermal stability of dried mushrooms was altered during the third stage (exothermic events, P3) in the temperature range of 106–172 °C in blanched samples, given the lower values of *T_o_* and *T_p_*. The lowest ΔH value among dried samples without pretreatment was found for FD-dried mushrooms (13.37 J/g), while values among pretreated samples were lower in case of UV exposed samples, especially when HAD or FD were applied (21.59–21.87 J/g). The next exothermic decomposition stage (P4) for the temperature range 274–370 °C, indicates that blanched samples, particularly dried by FD and HAD, reached higher values of *T_o_*, probably because these samples had already been subjected to heat-through water blanching-before drying. The enthalpy changes ΔH for this stage (P4) were significantly higher than those for the previous exothermic stage (P3). Considering the first two exothermic events (P3 and P4) and the *T_o_* and *T_p_* responses, it seems that drying mushrooms by CVD, especially without any pretreatment, produced more thermally stable products.

**Figure 8:**
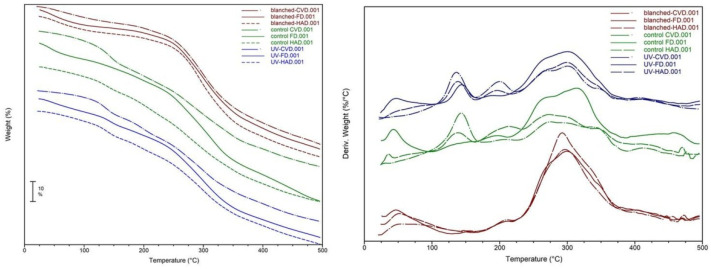
TG and DTG curves of *B. edulis* mushrooms subjected to different pretreatment and drying methods for the temperature range of 25–500 °C.

The TG and DTG curves of dried *B. edulis* mushrooms are presented in Figure 8.

The characteristics of the TG analysis of mushroom powders are illustrated in Table 4.

The TG-DTG analysis indicated weight loss during the whole temperature range, the derivative DTG curves showing the five identified peaks. The residual water removal from mushroom powders, as well as physical bond disruption [41] was evidenced at temperatures < 110 °C. At this stage, the DTG curves do not show peaks for UV-pretreated samples dried by HAD and CVD methods. The weight loss was <1% in case of control samples dried by HAD and CVD, while samples dried by FD and samples subjected to blanching showed higher values of Δm. The weight and volatile substance losses continued in the next stage, except for the blanched dried samples. The samples dried by CVD method showed higher values of Δm compared to those of HAD or FD. The TG determined initial (*T_i_*) and final (*T_f_*) temperature values were higher compared to peak values in DSC curves. Such differences between DSC and TG values have also been observed by other researchers, being explained by preventing the gas elimination from inside to outside of particles [44]. At temperatures > 180 °C, the next three peaks of the DTG curves, which correspond to the three exothermic peaks of the DSC curves, were attributed to the decomposition of polysaccharides (hemicellulose, cellulose) in mushrooms. Hemicellulose decomposes at lower temperatures than cellulose due to the lower degree of polymerization. Dorez et al. (2014) showed that the weight losses due to cellulose degradation, in the second exothermic stage (250–370 °C), are higher than those recorded in the first stage [46]. Our results are similar to their findings. Thus, the highest weight losses, at this stage, occurred in samples dried by FD method irrespective of the applied pretreatment. As shown in Figure 8 (DTG), the peaks recorded at temperature of ~300 °C corresponding to control dried samples or dried samples previously pretreated with UV-C, were broader. This might be explained by the degradation of polysaccharides/chitin, lignin, which occurs over a wide range of temperatures [42,46]. The third stage indicating the complete degradation of the mushroom biopolymers was not identified in dried samples previously subjected to water blanching.

## 3. Materials and Methods

### 3.1. Materials

Wild edible mushrooms (*B. edulis* L.) were manually harvested from Avrig forest, Sibiu, Romania, located at 45.661123 N, 24.445704 E and altitude of 500 m, during the mushroom season in 2021. All the mushroom samples were collected from the same natural forest (deciduous forest), with small distance between samples, on the same day. A total of 20 samples of the same species, *B. edulis* (cap and stipe) were sliced and divided into three portions of ~500 g. One portion was subjected to UV irradiation, a second one was blanched, while the third portion was kept as such. All samples were stored at −70 °C until analysis. Before drying, mushrooms were blended (Blendforce BL 438831) into a puree form such as to get a homogeneous sample containing cap and stipe. After drying, samples were grounded into powder using the knife mill (Grindomix GM 200, Retsch, Haan, Germany). The moisture content of fresh and dried samples was determined at 105 °C using the moisture analyzer (Mac 210/NP Radwag, Radom, Poland).

Chemical reagents of analytical grade without further purification were used.

### 3.2. Pretreatments

#### 3.2.1. UV-C Exposure

Fresh mushrooms were subjected to UV-C light at 254 nm, using a low pressure UV lamp with an illuminating intensity of 14 μW·cm^−2^ (6 KLU 254 + 366 nm, NeoLab, Heidelberg, Germany), in a closed box, for 30 min exposure time, at 20 cm exposure distance, conditions previously described by our group [15].

#### 3.2.2. Blanching

Fresh mushrooms were immersed into boiling water at 100 °C for 3 min, after which samples were drained on a stainless sieve and cooled.

All pretreated and control (untreated) samples were further dehydrated to an average moisture content of ~7% using different procedures, convective and vacuum, as described below.

### 3.3. Drying Procedures of Mushroom Blend

Convective drying using hot air was performed to efficiently dehydrate samples, this technique being widely used in food industry because of its simplicity and relatively low cost. Two vacuum drying methods were also performed, freeze drying as a friendly technique that preserves the nutritional quality of products, and centrifugal/rotational vacuum drying, which is an economical technique used especially for aqueous materials containing thermally unstable compounds.

HAD and CVD were conducted at 60 °C, a value at which drying time is lower than that of lower applied temperatures, and in agreement with most studies that have shown a decrease in the quality of products which have been dried at temperatures higher than 60 °C [47].

#### 3.3.1. Hot Air Drying (HAD)

Fresh mushroom blend of 200 g, distributed in Petri glass dishes of Φ7 cm in a layer of 1 cm thickness, was stored in a forced-air oven preheated at 60 °C (UFE 400 with forced air circulation, Memmert, Schwabach, Germany) at a maximum fan speed (100%). Aliquots were periodically removed for moisture analysis. Samples were dried until the final moisture content was <10%.

#### 3.3.2. Freeze Drying (FD)

Fresh mushroom blend of 100 g was dried under vacuum of 0.011 mbar at −60 °C using a freeze drier (Alpha 1-4 LDplus, Christ, Osterode am Harz, Germany). Drying was performed for 19 h until the moisture content reached 5.631% for control sample, 5.002% for UV-pretreated sample and 8.284% for blanched sample.

#### 3.3.3. Centrifugal Vacuum Drying (CVD)

Fresh mushroom blend of 200 g distributed in conical glass vials was subjected to rotational/centrifugal vacuum drying using a speed dry vacuum concentrator (RVC 2-18 CD plus, Christ, Osterode am Harz, Germany), at 60 °C and speed of 1200 rpm. Aliquots were periodically removed for moisture analysis. Drying was performed until the moisture content was <10%.

### 3.4. Quality Evaluations

Extracts of fresh and dried mushrooms were prepared in ethanol 70% (*v*/*v*) at a solvent/solid ratio of 10/1, at room temperature for 24 h. The mixture was then centrifuged at 4 °C at 8000 rpm for 10 min using the refrigerated centrifuge (Universal 320, Hettich, Berlin, Germany).

#### 3.4.1. Total Phenolic Content (TPC)

The total phenolic content was determined spectrophotometrically according to the Folin–Ciocalteu method [48]. The Specord 200 Plus UV-Vis spectrophotometer (Analytik Jena, Jena, Germany) was used. The results were expressed as mg gallic acid equivalents (GAE) per 100 g dry weight (DW).

#### 3.4.2. Total Antioxidant Activity (TAA)

The total antioxidant activity was determined spectrophotometrically according to the Ferric Reducing Antioxidant Power (FRAP) assay [49]. The results were expressed as mg ascorbic acid equivalents (AAE) per 100 g dry weight (DW).

#### 3.4.3. Rehydration Ratio

The rehydration ratio was determined by immersing 1 g of dried mushroom into boiling water for 10 min [50]. The mixture was then centrifuged at 7000 rpm and weighed. The rehydration ratio of the dried mushroom was calculated following the relation:

(1) $$\mathrm{Rehydration}\;\mathrm{ratio}\;=\frac{g\;\mathrm{of}\;\mathrm{rehydrated}\;\mathrm{sample}}{g\;\mathrm{of}\;\mathrm{dried}\;\mathrm{sample}}$$

#### 3.4.4. Color Measurements

The color of the acetone mushroom extracts was measured using the WinASpect Plus software version 4.0.0.0 (Analytik Jena, Jena, Germany) module of the Specord 200 Plus UV-Vis spectrophotometer (Analytik Jena, Jena, Germany) for the analysis of color according to DIN EN ISO 1164 (xyz, CIE, L*a*b) and ASTM E 313 (yellowness and whiteness indices) using the standard illuminant D65, field of view 2°.

The color was expressed as L* (lightness/darkness), a* (red/green) and b* (yellow/blue). The color difference between dried and raw mushrooms (ΔE*) was determined according to the equation:

(2) $$\Delta E{*}^{\;}=\sqrt{{\Delta L}^{*2}+{\Delta a}^{*2}+{\Delta b}^{*2}}$$

The whiteness and yellowness indices were also measured for standard CIE illuminant C and 2° field of view using the same software.

#### 3.4.5. SEM Analysis

Scanning electron micrographs were obtained with a Variable Pressure (VP) Field Emission Scanning Electron Microscope (Carl Zeiss, Oberkochen, Germany) operating at 30 kV for various magnifications, for microstructural analysis. In order to minimize beam damage and charging, the analysis of mushroom samples was performed in the VP mode and Backscattered Imaging.

#### 3.4.6. ATR-FTIR Analysis

Fourier Transform-Infrared (FT-IR) measurements were carried out using an ALPHA FT-IR spectrometer with the combined QuickSnapTM sampling modules and ZnSe ATR (Attenuated Total Reflectance) (Bruker, Ettlingen, Germany) with a resolution of 4 cm^−1^. An average of 32 scans was recorded in the ATR mode.

### 3.5. Thermal Analysis by Differential Scanning Calorimetry (DSC) and Thermogravimetry (TG)

The thermal behavior of fresh and dried mushrooms was evaluated by DSC analysis using the SDT Q600 calorimeter (TA Instruments) at a heating rate of 10 °C/min, under nitrogen flow of 20 mL/min. The instrument was calibrated using indium and zinc. Each sample weighing 5 ± 0.5 mg was put in an open DSC pan and heated up to 500 °C. The onset temperature (*T_o_*), the peak transition temperature (*T_p_*) and the enthalpy (the peak area of the DSC transition curve, ΔH) were calculated from the graphical representation of the heat flow against temperature. The software Universal Analysis 2000 supplied by TA Instruments was used to determine the values. The thermal weight loss (Δm), the initial temperature (*T_i_*) and the final temperature (*T_f_*) were determined by TG analysis using the same calorimeter. The first derivative of the TG curve was plotted (DTG) to determine the inflection points.

### 3.6. Statistical Analysis

The reported results are expressed as mean ± standard deviation (SD). In the present study, the data obtained from the three replications were analyzed using linear regressions and mathematical models to fit the data, and analysis of variance. An additional post-hoc test (Tukey test) was applied in the case of significant ANOVA. All analyses were performed using the R 4.1.1. statistical software (R Core Team 2021, https://www.r-project.org/ accessed on 17 May 2022). The mean values were considered significantly different at 95% confidence level (*p* < 0.05).

## 4. Conclusions

In the present study, the rehydration ratio, total phenolic content, antioxidant activity, color changes, microstructure, FTIR and thermal alterations were investigated in *B. edulis* mushroom puree subjected to different drying and pretreatment methods. The results showed that UV-C pretreatment of mushrooms proved to be a better alternative to water blanching, indicating good retention of phenolics and antioxidant activity and least total color changes. Structural changes of carbohydrates and proteins were confirmed by ATR-FTIR analysis. Centrifugal vacuum drying at 60 °C emerged as a new efficient mushroom drying technology in terms of phenolic content and antioxidant activity, color and thermal stability, but with low rehydration ratio of the final products. Freeze drying led to better rehydration ability and microstructure of dried samples, but prolonged drying time. Hot air drying at 60 °C proved to be an efficient method for dehydrating mushrooms, considering processing time, phenolic content and antioxidant activity.

## Figures and Tables

**Table 1:** Summary results of regression analysis for the effect of different pretreatment/drying methods on drying time of *B. edulis* mushrooms.

Sample Pretreatment	Drying Method/Regression	r^2^	F-Statistic	df	*p*-Value
Control	HAD~drying time	0.984	617.2	9	<0.05 ***
	CVD~drying time	0.798	40.67	9	0.00012
UV	HAD~drying time	0.972	352.40	9	<0.05 ***
	CVD~drying time	0.803	41.83	9	0.00011
Blanching	HAD~drying time	0.949	190.70	9	<0.05 ***
	CVD~drying time	0.724	27.26	9	0.0005

*** highly significant.

**Table 2:** The color characteristics and changes of *B. edulis* mushrooms under various pretreatment and drying methods.

Pretreatment	Drying	L*	a*	b*	ΔE*	Whiteness Index	Yellowness Index
Control	− Raw	91.67 ± 1.68	−11.38 ± 0.64	81.41 ± 1.85	-	−185.53	83.02
Blanching	− Raw	97.83 ± 2.21	−13.39 ± 1.43	42.14 ± 1.44	-	−104.16	52.58
UV	− Raw	96.80 ± 2.01	−12.39 ± 1.66	43.77 ± 1.11	-	−107.95	54.37
Control	HAD	81.58 ± 1.07	4.42 ± 0.47	79.02 ± 1.33	18.91	−145.06	85.71
	CVD	83.33 ± 1.87	0.57 ± 0.11	91.33 ± 2.04	17.63	−164.60	90.38
	FD	92.43 ± 1.30	−7.52 ± 0.75	28.57 ± 0.61	52.98	−49.09	40.03
Blanching	HAD	98.54 ± 3.22	−4.32 ± 0.70	9.64 ± 0.75	33.91	39.83	14.65
	CVD	97.64 ± 2.50	−10.30 ± 1.89	25.13 ± 0.86	17.41	−36.44	34.69
	FD	98.71 ± 2.25	−6.88 ± 0.53	15.95 ± 1.01	27.16	7.29	23.11
UV	HAD	94.04 ± 1.86	−5.16 ± 0.42	24.76 ± 0.97	20.52	−34.37	35.06
	CVD	96.90 ± 2.10	−3.65 ± 0.21	17.41 ± 0.82	27.78	−0.86	25.23
	FD	96.56 ± 2.05	−5.32 ± 0.78	15.73 ± 1.03	28.93	6.87	23.11

**Table 3:** Characteristic temperatures and enthalpy from the DSC investigation of dried *B. edulis* mushrooms.

Peak	DSC Values	Sample (Pretreatment and Drying Methods)								
		Control HAD	Control CVD	Control FD	Blanched HAD	Blanched CVD	Blanched FD	UV-HAD	UV-CVD	UV-FD
P1	*T_p_*, °C	57.08	46.73	46.26	50.76	42.11	51.85	n.d.	42.27	47.66
P2	*T_o_*, °C	113.55	106.89	n.d.	n.d.	n.d.	n.d.	114.38	121.19	n.d.
	*T_p_*, °C	130.12	145.30	n.d.	n.d.	n.d.	n.d.	136.72	141.21	n.d.
	Δ*H*, J/g	6.18	34.78	n.d.	n.d.	n.d.	n.d.	11.65	28.03	n.d.
P3	*T_o_*, °C	133.59	150.02	123.66	105.67	118.44	106.34	144.72	146.56	136.97
	*T_p_*, °C	166.20	172.02	143.05	143.36	157.59	145.42	167.17	168.58	167.50
	Δ*H*, J/g	22.20	20.48	13.37	89.53	40.25	147.90	21.59	26.11	21.87
P4	*T_o_*, °C	299.56	300.68	293.13	318.95	295.97	325.32	289.52	274.15	292.75
	*T_p_*, °C	331.84	353.12	333.96	369.45	343.67	363.58	335.73	330.84	332.30
	Δ*H*, J/g	79.24	324.20	580.70	205.70	227.70	171.00	215.80	113.20	190.20
P5	*T_o_*, °C	465.52	n.d.	408.24	468.02	450.09	444.06	n.d.	407.49	455.08
	*T_p_*, °C	474.30	n.d.	464.68	472.96	455.86	455.26	n.d.	435.81	462.73
	Δ*H*, J/g	9.00	n.d.	195.80	4.01	7.42	57.69	n.d.	15.22	7.31

n.d. = not detected.

**Table 4:** Results of TG investigation of *B. edulis* mushrooms subjected to different pretreatment and drying methods.

Peak	TG Values	Sample (Pretreatment and Drying Methods)								
		Control HAD	Control CVD	Control FD	Blanched HAD	Blanched CVD	Blanched FD	UV-HAD	UV-CVD	UV-FD
P1	*T_i_* (°C)	32.91	26.89	35.84	40.18	40.54	38.73	n.d.	n.d.	35.29
	*T_f_* (°C)	37.57	28.52	61.18	82.15	110.83	78.52	n.d.	n.d.	66.24
	Δm, %	0.87	0.69	8.01	7.51	8.48	8.35	n.d.	n.d.	6.63
P2	*T_i_* (°C)	120.71	111.35	n.d.	n.d.	n.d.	n.d.	124.04	118.89	130.11
	*T_f_* (°C)	150.34	155.63	n.d.	n.d.	n.d.	n.d.	150.70	147.64	154.27
	Δm, %	8.79	19.88	n.d.	n.d.	n.d.	n.d.	9.41	12.89	8.21
P3	*T_i_* (°C)	188.63	194.75	180.47	191.45	190.43	190.26	181.40	183.34	179.36
	*T_f_* (°C)	221.28	214.23	204.45	213.97	213.90	210.54	206.33	211.06	192.38
	Δm, %	11.43	6.80	6.48	2.97	4.21	3.24	7.14	9.51	3.60
P4	*T_i_* (°C)	251.97	250.02	243.37	264.16	260.13	248.19	257.58	258.35	249.62
	*T_f_* (°C)	309.78	351.51	341.85	335.53	336.17	350.46	335.38	330.31	339.71
	Δm, %	24.17	38.54	57.57	47.57	47.02	57.61	34.31	30.66	41.34
P5	*T_i_* (°C)	347.88	401.39	426.14	n.d.	n.d.	n.d.	400.31	397.27	403.40
	*T_f_* (°C)	364.61	434.66	472.37	n.d.	n.d.	n.d.	435.28	439.17	435.23
	Δm, %	10.36	7.23	14.10	n.d.	n.d.	n.d.	8.11	8.87	5.76

n.d. = not detected.

## Data Availability

The data presented in this study are available on request from the corresponding author.

## Sample Availability

Samples of the compounds are not available from the authors.

## References

[1] Cheung P.C.K. (2008). Mushrooms as Functional Foods.

[2] Argyropoulos D., Khan M.T., Müller J. (2011). Effect of Air Temperature and Pre-Treatment on Color Changes and Texture of Dried *Boletus Edulis* Mushroom. Dry. Technol..

[3] Novakovic S. (2021). The Potential of the Application of *Boletus Edulis, Cantharellus Cibarius* and *Craterellus Cornucopioides* in Frankfurters: A Review. IOP Conf. Ser. Earth Environ. Sci..

[4] Castellanos-Reyes K., Villalobos-Carvajal R., Beldarrain-Iznaga T. (2021). Fresh Mushroom Preservation Techniques. Foods.

[5] Zhang K., Pu Y.-Y., Sun D.-W. (2018). Recent Advances in Quality Preservation of Postharvest Mushrooms (Agaricus Bisporus): A Review. Trends Food Sci. Technol..

[6] Chakraverty A., Mujumdar A.S., Ramaswamy H.S. (2003). Handbook of Postharvest Technology: Cereals, Fruits, Vegetables, Tea, and Spices.

[7] Jaworska G., Pogoń K., Bernaś E., Skrzypczak A. (2014). Effect of Different Drying Methods and 24-Month Storage on Water Activity, Rehydration Capacity, and Antioxidants in *Boletus Edulis* Mushrooms. Dry. Technol..

[8] Ratti C. (2001). Hot Air and Freeze-Drying of High-Value Foods: A Review. J. Food Eng..

[9] Yapar S., Helvaci S.Ş., Peker S. (1990). Drying behavior of mushroom slices. Dry. Technol..

[10] Pasban A., Mohebbi M., Pourazarang H., Varidi M. (2014). Effects of Endemic Hydrocolloids and Xanthan Gum on Foaming Properties of White Button Mushroom Puree Studied by Cluster Analysis: A Comparative Study. J. Taibah Univ. Sci..

[11] Dziki D. (2020). Recent Trends in Pretreatment of Food before Freeze-Drying. Processes.

[12] Ocoró-Zamora M.U., Ayala-Aponte A.A. (2013). Influence of Thickness on the Drying of Papaya Puree (*Carica Papaya* L.) through Refractance Window Technology. Dyna.

[13] Llavata B., García-Pérez J.V., Simal S., Cárcel J.A. (2020). Innovative Pre-Treatments to Enhance Food Drying: A Current Review. Curr. Opin. Food Sci..

[14] Deng L.-Z., Mujumdar A.S., Zhang Q., Yang X.-H., Wang J., Zheng Z.-A., Gao Z.-J., Xiao H.-W. (2019). Chemical and Physical Pretreatments of Fruits and Vegetables: Effects on Drying Characteristics and Quality Attributes—A Comprehensive Review. Crit. Rev. Food Sci. Nutr..

[15] Oancea S., Popa M., Răcuciu M., Oancea S., Popa-Vecerdea F.M., Răcuciu M. (2021). Effects of non-thermal postharvest irradiation of dried mushrooms on their antioxidant content and activity. Rom. Rep. Phys..

[16] Xiao H.-W., Pan Z., Deng L.-Z., El-Mashad H.M., Yang X.-H., Mujumdar A.S., Gao Z.-J., Zhang Q. (2017). Recent Developments and Trends in Thermal Blanching—A Comprehensive Review. Inf. Process. Agric..

[17] Thamkaew G., Sjöholm I., Galindo F.G. (2021). A Review of Drying Methods for Improving the Quality of Dried Herbs. Crit. Rev. Food Sci. Nutr..

[18] Jambrak A.R., Mason T.J., Paniwnyk L., Lelas V. (2007). Accelerated Drying of Button Mushrooms, Brussels Sprouts and Cauliflower by Applying Power Ultrasound and Its Rehydration Properties. J. Food Eng..

[19] Kaveh M., Abbaspour-Gilandeh Y., Fatemi H., Chen G. (2021). Impact of Different Drying Methods on the Drying Time, Energy, and Quality of Green Peas. J. Food Process. Preserv..

[20] Argyropoulos D., Heindl A., Müller J. Evaluation of processing parameters for hot-air drying to obtain high quality dried mushrooms in the Mediterranean region. Proceedings of the Conference on International Research on Food Security, Natural Resource Management and Rural Development, University of Hohenheim.

[21] Wang H.C., Zhang M., Adhikari B. (2015). Drying of Shiitake Mushroom by Combining Freeze-Drying and Mid-Infrared Radiation. Food Bioprod. Process..

[22] Piskov S., Timchenko L., Grimm W.-D., Rzhepakovsky I., Avanesyan S., Sizonenko M., Kurchenko V. (2020). Effects of Various Drying Methods on Some Physico-Chemical Properties and the Antioxidant Profile and ACE Inhibition Activity of Oyster Mushrooms (Pleurotus Ostreatus). Foods.

[23] Priyadarsini D., Rayaguru K., Misra S., Dash S.K. (2022). Effect of Drying Techniques on Physicochemical Properties of Oyster Mushroom (Pleurotus Sajor-Caju). J. Food Process. Preserv..

[24] Ren S., Zheng E., Zhao T., Hu S., Yang H. (2022). Evaluation of physicochemical properties, equivalent umami concentration and antioxidant activity of *Coprinus comatus* prepared by different drying methods. LWT-Food Sci. Technol..

[25] Nagy M., Socaci S., Tofană M., Biris-Dorhoi E.S., Țibulcă D., Salanță L., Petruț G. (2017). Chemical composition and bioactive compounds of some wild edible mushrooms. Bull. UASVM Food Sci. Technol..

[26] Kuka M., Cakste I. Bioactive compounds in Latvian wild edible mushroom Boletus edulis. Proceedings of the 6th Baltic Conference on Food Science and Technology “Innovations for Food Science and Production: FOODBALT-2011”.

[27] Fogarasi M., Socaciu M.I., Sălăgean C.D., Ranga F., Fărcaș A.C., Socaci S.A., Socaciu C., Țibulcă D., Fogarasi S., Semeniuc C.A. (2021). Comparison of Different Extraction Solvents for Characterization of Antioxidant Potential and Polyphenolic Composition in *Boletus edulis* and *Cantharellus cibarius* Mushrooms from Romania. Molecules.

[28] Jaworska G., Pogoń K., Skrzypczak A., Bernaś E. (2015). Composition and Antioxidant Properties of Wild Mushrooms *Boletus edulis* and *Xerocomus badius* Prepared for Consumption. J. Food Sci. Technol..

[29] Kolayli S., Sahin H., Aliyazicioglu R., Sesli E. (2012). Phenolic Components and Antioxidant Activity of Three Edible Wild Mushrooms from Trabzon, Turkey. Chem. Nat. Compd..

[30] Mutukwa I.B., Hall III C.A., Cihacek L., Lee C.W. (2019). Evaluation of Drying Method and Pretreatment Effects on the Nutritional and Antioxidant Properties of Oyster Mushroom (Pleurotus Ostreatus). J. Food Process. Preserv..

[31] Abascal K., Ganora L., Yarnell E. (2005). The Effect of Freeze-Drying and Its Implications for Botanical Medicine: A Review. Phytother. Res..

[32] Fang T.T., Footrakul P., Luh B.S. (1971). Effects of blanching, chemical treatments and freezing methods on quality of freeze-dried mushrooms. J. Food Sci..

[33] Lv G., Zhang Z., Pan H., Fan L. (2014). Effect of Physical Modification of Mushroom (A. Chaxingu) Powders on Their Physical and Chemical Properties. Food Sci. Technol. Res..

[34] Lewicki P.P., Pawlak G. (2003). Effect of Drying on Microstructure of Plant Tissue. Dry. Technol..

[35] Koutrotsios G., Tagkouli D., Bekiaris G., Kaliora A., Tsiaka T., Tsiantas K., Chatzipavlidis I., Zoumpoulakis P., Kalogeropoulos N., Zervakis G.I. (2022). Enhancing the Nutritional and Functional Properties of *Pleurotus citrinopileatus* Mushrooms through the Exploitation of Winery and Olive Mill Wastes. Food Chem..

[36] Bekiaris G., Tagkouli D., Koutrotsios G., Kalogeropoulos N., Zervakis G.I. (2020). Pleurotus Mushrooms Content in Glucans and Ergosterol Assessed by ATR-FTIR Spectroscopy and Multivariate Analysis. Foods.

[37] Ghosh R.K., Kar T., Dutta B., Pathak A., Rakshit R., Basak R., Das A., Waheeda K., Basak P., Bhattacharyya M. (2018). Aberration in the Structural Paradigm of Lens Protein α Crystallin by UV-C Irradiation. Appl. Biol. Chem..

[38] Cruz-Angeles J., Martínez L.M., Videa M. (2015). Application of ATR-FTIR Spectroscopy to the Study of Thermally Induced Changes in Secondary Structure of Protein Molecules in Solid State. Biopolymers.

[39] Forero D.P., Carriazo J.G., Osorio C. (2016). Effect of Different Drying Methods on Morphological, Thermal, and Biofunctional Properties of Lulo (*Solanum Quitoense* Lam.) Fruit Powders. Dry Technol..

[40] Ju Z.Y., Hettiarachchy N., Kilara A. (1999). Thermal Properties of Whey Protein Aggregates. J. Dairy Sci..

[41] Shakir M.A., Azahari B., Yusup Y., Yhaya M.F., Salehabadi A., Ahmad M.I. (2020). Preparation and Characterization of Mycelium as a Bio-Matrix in Fabrication of Bio-Composite. J. Adv. Res. Fluid Mech. Therm. Sci..

[42] Ospina Álvarez S.P., Ramírez Cadavid D.A., Escobar Sierra D.M., Ossa Orozco C.P., Rojas Vahos D.F., Zapata Ocampo P., Atehortúa L. (2014). Comparison of Extraction Methods of Chitin from *Ganoderma Lucidum* Mushroom Obtained in Submerged Culture. BioMed Res. Int..

[43] Kalač P. (2009). Chemical Composition and Nutritional Value of European Species of Wild Growing Mushrooms: A Review. Food Chem..

[44] Huang J., Liu J., Chen J., Xie W., Kuo J., Lu X., Chang K., Wen S., Sun G., Cai H. (2018). Combustion Behaviors of Spent Mushroom Substrate Using TG-MS and TG-FTIR: Thermal Conversion, Kinetic, Thermodynamic and Emission Analyses. Bioresour. Technol..

[45] Dinani T.S., Hamdami N., Shahedi M., Havet M., Queveau D. (2015). Influence of the Electrohydrodynamic Process on the Properties of Dried Button Mushroom Slices: A Differential Scanning Calorimetry (DSC) Study. Food Bioprod. Process..

[46] Dorez G., Ferry L., Sonnier R., Taguet A., Lopez-Cuesta J.-M. (2014). Effect of Cellulose, Hemicellulose and Lignin Contents on Pyrolysis and Combustion of Natural Fibers. J. Anal. Appl. Pyrolysis.

[47] Kotwaliwale N., Bakane P., Verma A. (2007). Changes in Textural and Optical Properties of Oyster Mushroom during Hot Air Drying. J. Food Eng..

[48] Singleton V.L., Rossi J.A. (1965). Colorimetry of Total Phenolics with Phosphomolybdic-Phosphotungstic Acid Reagents. Am. J. Enol. Vitic..

[49] Benzie I.F.F., Strain J.J. (1996). The Ferric Reducing Ability of Plasma (FRAP) as a Measure of “Antioxidant Power”: The FRAP Assay. Anal. Biochem..

[50] Kantrong H., Tansakul A., Mittal G.S. (2014). Drying Characteristics and Quality of Shiitake Mushroom Undergoing Microwave-Vacuum Drying and Microwave-Vacuum Combined with Infrared Drying. J. Food Sci. Technol..

